# Structural importance of the C-terminal region in pig aldo-keto reductase family 1 member C1 and their effects on enzymatic activity

**DOI:** 10.1186/s12900-014-0028-7

**Published:** 2015-01-13

**Authors:** Minky Son, Chanin Park, Seul Gi Kwon, Woo Young Bang, Sam Woong Kim, Chul Wook Kim, Keun Woo Lee

**Affiliations:** Division of Applied Life Science (BK21 Plus), Systems and Synthetic Agrobiotech Center (SSAC), Plant Molecular Biology and Biotechnology Research Center (PMBBRC), Research Institute of Natural Science (RINS), Gyeongsang National University (GNU), 501 Jinju-daero, Jinju, 660-701 Republic of Korea; Swine Science and Technology Center, Gyeongnam National University of Science & Technology, Jinju, 660-758 Korea; National Institute of Biological Resources, Environmental Research Complex, Incheon, 404-708 Korea

**Keywords:** Aldo-keto reductase, Homology modeling, Molecular dynamic simulation, NADPH-dependent reduction, Steroid hormone

## Abstract

**Background:**

Pig aldo-keto reductase family 1 member C1 (AKR1C1) belongs to AKR superfamily which catalyzes the NAD(P)H-dependent reduction of various substrates including steroid hormones. Previously we have reported two paralogous pig AKR1C1s, wild-type AKR1C1 (C-type) and C-terminal-truncated AKR1C1 (T-type). Also, the C-terminal region significantly contributes to the NADPH-dependent reductase activity for 5α-DHT reduction. Molecular modeling studies combined with kinetic experiments were performed to investigate structural and enzymatic differences between wild-type AKR1C1 C-type and T-type.

**Results:**

The results of the enzyme kinetics revealed that *V*_max_ and *k*_cat_ values of the T-type were 2.9 and 1.6 folds higher than those of the C-type. Moreover, catalytic efficiency was also 1.9 fold higher in T-type compared to C-type. Since x-ray crystal structures of pig AKR1C1 were not available, three dimensional structures of the both types of the protein were predicted using homology modeling methodology and they were used for molecular dynamics simulations. The structural comparisons between C-type and T-type showed that 5α-DHT formed strong hydrogen bonds with catalytic residues such as Tyr55 and His117 in T-type. In particular, C3 ketone group of the substrate was close to Tyr55 and NADPH in T-type.

**Conclusions:**

Our results showed that 5α-DHT binding in T-type was more favorable for catalytic reaction to facilitate hydride transfer from the cofactor, and were consistent with experimental results. We believe that our study provides valuable information to understand important role of C-terminal region that affects enzymatic properties for 5α-DHT, and further molecular mechanism for the enzyme kinetics of AKR1C1 proteins.

## Background

The aldo-keto reductase (AKR) superfamily is mostly comprised of monomeric oxidoreductases that catalyze NAD(P)H-dependent reductions of a wide range of aldehydes and ketones including steroids, carbohydrates, bile acids, and prostaglandins [1,2]. The AKRs have been classified into 14 families (AKR1 to AKR14) and AKR1 family have been further divided into 6 subfamilies (AKR1A to AKR1G) [3,4]. Among the subfamilies, AKR1C enzymes are known as hydroxysteroid dehydrogenases (HSDs) which play a pivotal role in metabolism and regulation of steroid hormones such as progesterone, 5α-dihydrotestosterone (DHT), and testosterone. Pig aldo-keto reductase family 1 member C1 (AKR1C1) shows both 3α- and 20α-HSD activities and also plays a crucial role in progesterone metabolism, maintenance of pregnancy, and hormone regulation during the estrous cycle [5]. It officially named as AKRlCL1 (aldo-keto reductase family 1, member C-like 1), consists of 14 amino acid residues longer than that of general AKR1C1 [3]. The longer amino acid residues have been reported to alter enzymatic activities of several steroid hormones [3]. The structures of AKRs have the (α/β)_8_-barrel or TIM-barrel motif and three conserved loop regions, loop A, B, and C, which are related with steroid hormone specificity [4]. The enzymes catalyze an ordered bisequential kenetic process in which binding of cofactor is obligatory for the reaction [6,7]. The nicotinamide group of NADPH cofactor lies in *anti*-conformation with respect to the ribose group, so that 4-pro-R-hydride is transferred from the cofactor to the 3-ketosteroid substrate [2,6,8]. The hydride transfer is mediated by a highly conserved catalytic tetrad consisting of Asp50, Tyr55, Lys84, and His117, where Tyr 55 acts as the general acid/base [9-11]. Recently we have identified two paralogous pig AKR1C1s with or without C-terminal region (R320 to L337) which was truncated by a non-synonymous variation [3]. Also, the C-terminal region significantly affects the NADPH-dependent reductase activity for 5α-DHT reduction [3].

In this study, we performed molecular modeling studies combined with kinetic experiments to examine structural difference between wild-type AKR1C1 (C-type) and C-terminal-truncated AKR1C1 (T-type) for 5α-DHT. Since there was no available experimental structure of pig AKR1C1, we have carried out homology modeling to build 3D structure models of the both types, which were used for molecular dynamics (MD) simulation study. Our findings provide structural insights into important role of C-terminal region of the enzyme. It can be helpful for understanding different enzymatic properties for 5α-DHT between C-type and T-type.

## Methods

### Materials

The following chemicals were used in the experiments; 5α-dihydrotestosterone (5α-DHT), methylglyoxal, 9,10-phenanthrenequinone and hydrindantin were purchased from Sigma (St. Louis, MO), and Ni-NTA chelating agarose CL-6B was purchased from Peptron company (Promega corporaton, USA). Bio-Rad Bradford Protein assay kit was purchased from (Bio-Rad Laboratories, Inc (South Korea). The others, including Na2HPO4, NaH2PO4, NaCl, bovine serum albumin (BSA), and imidazole, were purchased from Sigma (St. Louis, MO).

### Recombinant protein purification

In previous study [3], two types of pPROEX HTb-AKR1CL1 clones were constructed for the production of his-tagged fusion proteins for C-type and T-type. They were used for the IPTG-induced expression of each of the clones in *E. coli* BL21. In this study, the IPTG-induced proteins were subjected to the affinity chromatography using Ni-NTA agarose, according to manufacturer's manual (Peptron, Daejon, Korea). Briefly, basal buffer for protein purification was prepared by 50 mM sodium phosphate buffer, pH8.0, and 500 mM NaCl. Imidazole (Sigma, USA) was added to required concentration according to purification manual with NTA Chelating Agarose CL-6B (Promega corporation, USA). The overexpressed cells were precipitated by centrifugation, and suspended by binding buffer including 5 mM imidazole. The collected cells were lyzed by SONICS Vibracell VCX750 Ultrasonic Cell Disruptor, which was done twice by conditions as following; 5 min by 2 sec interval of on/off and 35% amplitude during ice cooling. The supernatant to obtain water-soluble protein was collected from the cells treated by centrifugation for 30 min at 10,000 rpm, 4°C. Purification of the protein was done by NTA Chelating Agarose CL-6B (Promega corporation, USA) according to manufacturer’s directions. The purified recombinant proteins were concentrated by Ultrafree-0.5 Centrifugal Filter Device (Millipore Corporation, Germany). The concentrated proteins were quantified by Bio-Rad Bradford Protein assay kit (Bio-Rad Laboratories, Inc., Korea) by OD595 nm in wavelength. The purified proteins were added with 50% glycerol and 50 mM Sodium Phosphate Buffer (pH 6.4) for long-term storage at −20°C.

### Measurement of NADPH-dependent carbonyl reductase activity

The reductase activity was measured under conditions described previously [12]. Reaction mixtures included 60 mM sodium phosphate (pH 6.5), purified recombinant proteins such as C-type and T-type, 0.1 mM NADPH and 0.1 mM substrates (the reproductive steroid hormones indicated above) and were incubated in a total volume of 0.5 ml at 37°C. The assay of reductase activity was spectrophotometrically carried out by monitoring the decrease in absorbance at 340 nm with time.

### Statistical analysis

To determine kinetic parameters with a Michaelis–Menten plot, the data were analyzed by nonlinear regression using GraphPad prism 6 software (GraphPad Software Inc., San Diego, CA). Enzyme concentrations of 39 and 38.9 nmol/mg were used for the calculation of turnover rates (*k*_cat_) for C-type and T-type, respectively. The significant differences were analyzed by Student’s t-test (*p* < 0.01 or *p* < 0.05) using the above software. The results are expressed as means ± standard errors (S.E.) of at least 3 independent experiments.

### Homology modeling

The sequence of pig AKR1C1 consisting of 337 amino acids, was obtained from UniProtKB (http://www.uniprot.org/) (accession no. Q1KLB4). In order to build a structure model of pig AKR1C1 homology modeling was conducted using Phyre2 server (Protein Homology/analogY Recognition Engine V 2.0) with intensive modeling mode [13], which the server utilizes multiple templates and *ab initio* techniques to predict 3D structure model. The generated homology model was subjected to energy minimization to refine the model as well as to reduce steric clashes. The minimization with the steepest descent algorithm for 10,000 steps was carried out by GROMACS 4.5.3 package [14,15] with CHARMM27 force field. The stereochemical quality of the model was assessed by PROCHECK [16], ProSA [17,18], and ERRAT [19]. All other analyses including multiple sequence alignment were done by Discovery Studio v3.1 (DS).

### Molecular docking calculation

AKR1C1 C-type and T-type in complex with NADPH were subjected to molecular docking calculation. The structure of T-type was prepared by deleting the C-terminal region (R320 to L337) from C-type. The coordinates of the cofactor were taken from the structure of human AKR1C3 (PDB: 1S1P). The substrate, 5α-DHT, was downloaded from PubChem Compound Database (CID: 10635) [20]. Then 5α-DHT was subjected to energy minimization with CHARMm force field and implicit solvent model using DS. The binding pose of 5α-DHT was predicted using GOLD v 5.0.1 (Genetic Optimization for Ligand Docking) [21,22] which uses genetic algorithm (GA) for docking flexible ligands in the binding site of the protein. The binding site was assigned through *Define and Edit Binding Site* tool in DS. All residues within the radius of 5 Å of the center of binding sphere were included in the calculation and the number of GA runs was set to 100. All other parameters were used as their default values. The docking poses were ranked based on GOLD fitness score and top solution was selected as initial conformation for MD simulation.

### Molecular dynamics simulation

MD simulations for C-type and T-type in complex with NADPH and 5α-DHT were performed using GROMACS 4.5.3 with CHARMM27 force field. Topology files for the ligands were obtained from SwissParam server [23]. At the beginning, protonation states of the ionizable residues were set at pH7. A water box with the size of 1.5 nm from the protein surface was created to make an aqueous environment, and immersed using explicit TIP3P water model [24]. The size of the system was 6.05 × 5.78 × 5.90 nm for C-type and 6.03 × 5.77 × 5.03 nm for T-type, respectively. Several water molecules were replaced with sodium ions to neutralize the system. Energy minimization for 10,000 steps was executed using steepest descent algorithm until the maximum force lower than 1000 kJ/mol. After minimization, the systems were subjected to 100 ps NVT equilibration at 300 K and then 100 ps NPT equilibration at 300 K and 1 bar of pressure. The equilibrated systems were used in 20 ns production runs under NPT ensemble. A constant temperature and pressure were kept using V-rescale thermostat [25] and Parrinello-Rahman barostat [26,27]. During the simulation, LINCS [28,29] and SETTLE [30] algorithms were used to constrain all bond lengths and the geometry of water molecules, respectively. Short-range interactions were treated with the cut-off value of 1.2 nm and long-range electrostatic interactions were calculated by applying particle mesh Ewald (PME) method [31,32]. The periodic boundary conditions were adopted to avoid edge effects. A grid spacing of 0.12 nm was applied for fast Fourier transform calculations. We repeated the simulations two times under the same conditions except that the simulation time was 10 ns. All simulations were performed with the time step of 2 fs and the coordinates were saved every 1 ps for analyses.

## Results and discussion

### The C-terminal region in AKR1C1 alters significantly the enzymatic properties to 5α-DHT

AKR1C1 exhibits broadly enzymatic activities to various steroid hormones [33]. Among steroid hormones, AKR1C1 originated from human, previously named as 20α-hydroxysteroid dehydrogenase, detects specifically to progesterone with high activity [33]. A variant truncated at the C-terminus of pig AKR1C1 was employed in this study. In our previous study, we found a new novel single nucleotide variant (SNV) truncated in C-terminus, where the SNV is a nonsense mutant lacking 18 amino acid residues (R320 to L337) in C-terminus [3]. During the evaluation of enzymatic activities with different steroid hormones, differential activities between AKR1C1 C- and T-types were shown, 5α-DHT being anyway the preferred substrate for both of them [3]. Therefore, 5α-DHT was employed for enzymatic kinetics in this study.

**Figure 1:**
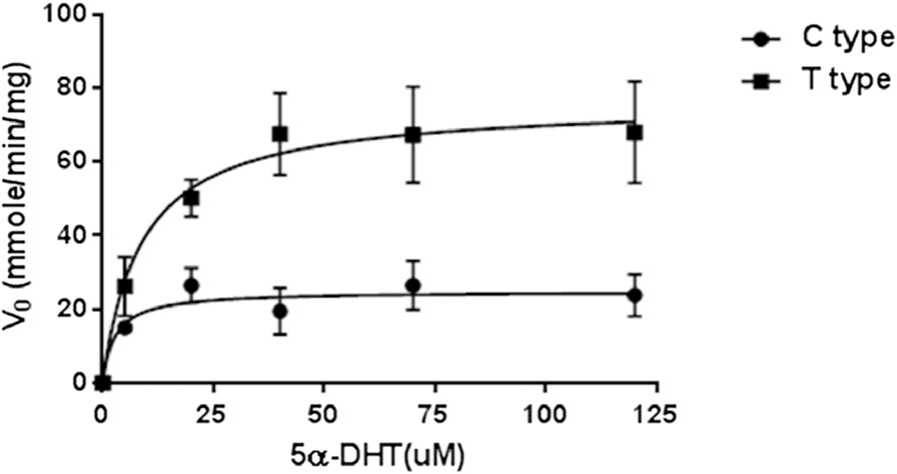
**A Michaelis-Menten plot from measurement of the NADPH-dependent reduction of 5α-DHT by recombinant AKR1C1 (WT) or AKR1C1 (ΔC term).** The data, obtained from measurement of the NADPH-dependent reduction of 5α-DHT by recombinant AKR1C1 (WT) and AKR1C1 (ΔC term), were analyzed by nonlinear regression using GraphPad prism 6 software (GraphPad Software Inc., San Diego, CA). Each spot represents the mean ± S.E. (n=3).

In order to analyze enzymatic activity of AKR1C1s, the enzymes were cloned into overexpression vector and then purified to homogeneity by affinity chromatography. The purified AKR1C1s were applied for enzymatic kinetics with substrate 5α-DHT and cofactor NADPH. The *V*_max_ value of AKR1C1 T-type was 2.9 fold higher than that of C-type, but *K*_m_ value was lower 1.6 fold (Figure 1 and Table 1). Furthermore, the values of *k*_cat_ and catalytic efficiency of the T-type were 2.9 and 1.9 folds higher than those of C-type. These results suggest that C-terminal truncated AKR1C1 improves the values of *V*_max,_*k*_cat_ and catalytic efficiency.

**Table 1:** **Kinetic parameters for 5α-DHT reduction measured by spectrophotometer**

**Enzyme**	***V***_***max***_	***K***_***m***_	***K***_***cat***_	***K***_***cat***_ ***/K***_***m***_
	**(nmol/min per mg)**	**(μM)**	**(s** ^**−1**^ **)**	**(s** ^**−1**^ **M** ^**−1**^ **)**
AKR1C1 C-type	25.74 ± 2.338	4.978 ± 3.091	0.011 ± 0.000999	2,210 ± 323.196
AKR1C1 T-type	75.5 ± 5.2	7.736 ± 2.816	0.0340 ± 0.00234	4,340 ± 830.966

Each value indicates mean ± SEM (n = 3).

Kinetic parameters were determined using data in Figure 1 through GraphPad prism 6 software (GraphPad Software Inc., San Diego, CA).

### The structure prediction of pig AKR1C1 using homology modeling

**Figure 2:**
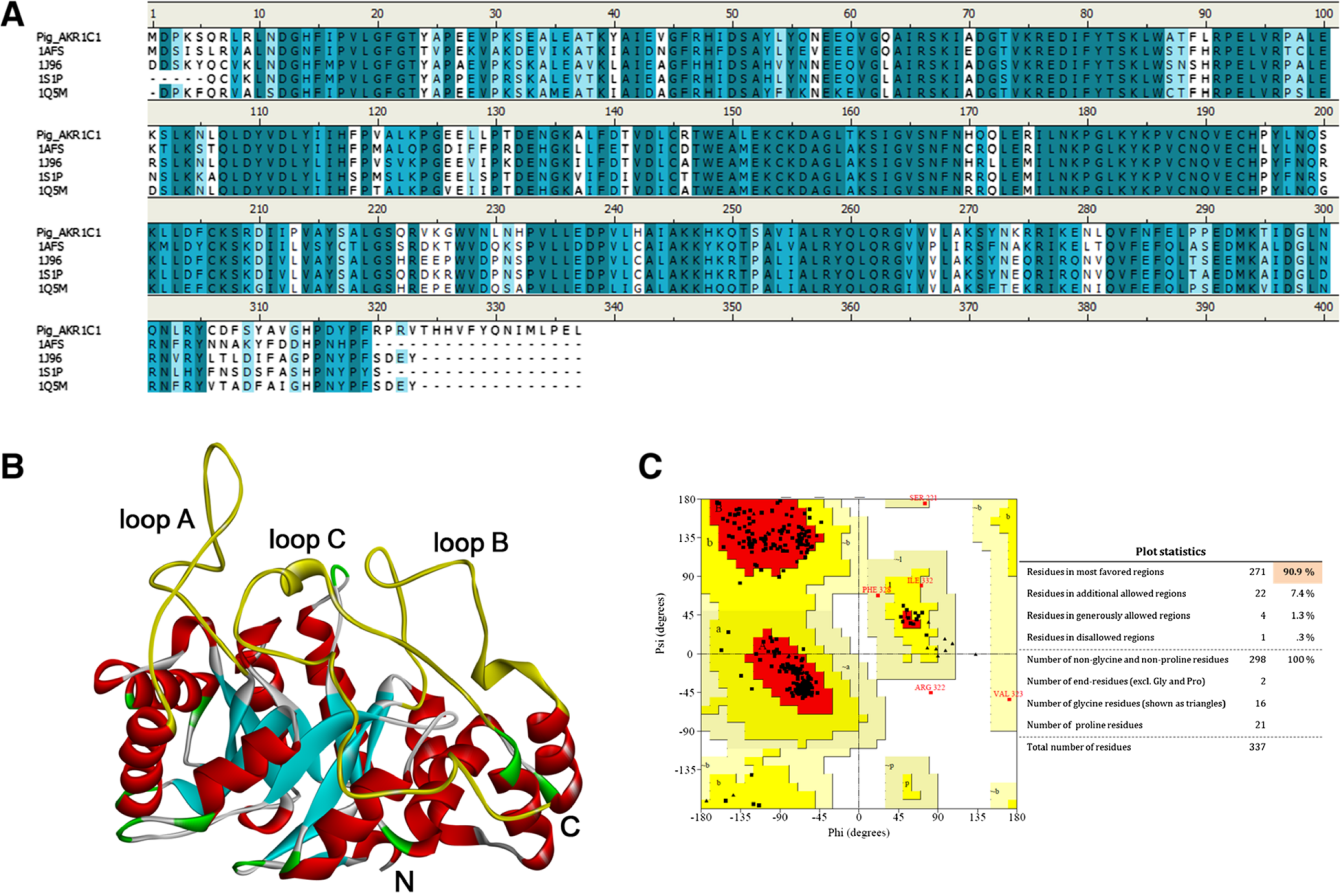
**The result of homology modeling. A** Multiple sequence alignment of pig AKR1C1 with rat AKR1C9 (PDB: 1AFS), human AKR1C2 (PDB: 1 J96), human AKR1C3 (PDB: 1S1P), and rabbit AKR1C5 (PDB: 1Q5M). **B** The 3D structure model of pig AKR1C1. Protein is represented as cartoon model and colored by secondary structure. Loop A, loop B, and loop C are displayed as yellow. **C** Ramachandran plot of pig AKR1C1 structure.

Since crystal structure of pig AKR1C1 has not been determined yet, we have constructed the 3D structure model using four structures as templates; rat AKR1C9 (PDB: 1AFS), human AKR1C2 (PDB: 1 J96), human AKR1C3 (PDB: 1S1P), and rabbit AKR1C5 (PDB: 1Q5M). The multiple sequence alignment with the four templates revealed that catalytic tetrad of Asp50, Tyr55, Lys84, and His117 were conserved and they have high sequence identity and similarity with each template; 71.5% and 85.6% between Pig AKR1C1 and rat AKR1C9, 75.5% and 88.2% between Pig AKR1C1 and human AKR1C2, 75.9% and 88.8% between Pig AKR1C1 and human AKR1C3, 76.2% and 88.5% between Pig AKR1C1 and rabbit AKR1C5, respectively (Figure 2A). Since there was no proper structural information for 14 residues at the end of the C-terminal region of AKR1C1, the region was modeled by *ab initio* method. The homology model for pig AKR1C1 was refined through the energy minimization and it showed conserved loop regions which are structural features of AKR superfamily (Figure 2B). The stereochemical quality of the generated model was evaluated using three programs. Ramachandran plot obtained from PROCHECK showed that 90.9% of residues were in most favored regions and only one residue was in disallowed region (Figure 2C). Overall quality factor scores calculated from ERRAT and ProSA were 90.49 and −11.04, respectively.

### Binding mode of 5α-DHT in the active site of AKR1C1 C-type and T-type

**Figure 3:**
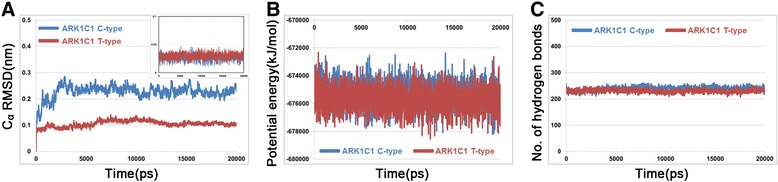
**The overall stability of MD simulation. A** RMSD for protein C_α_ atoms and 5α-DHT (inserted graph), **B** potential energy, and **C** the number of intra-hydrogen bonds were calculated during 20 ns simulation time. AKR1C1 C-type and T-type are represented as blue and red lines, respectively.

**Figure 4:**
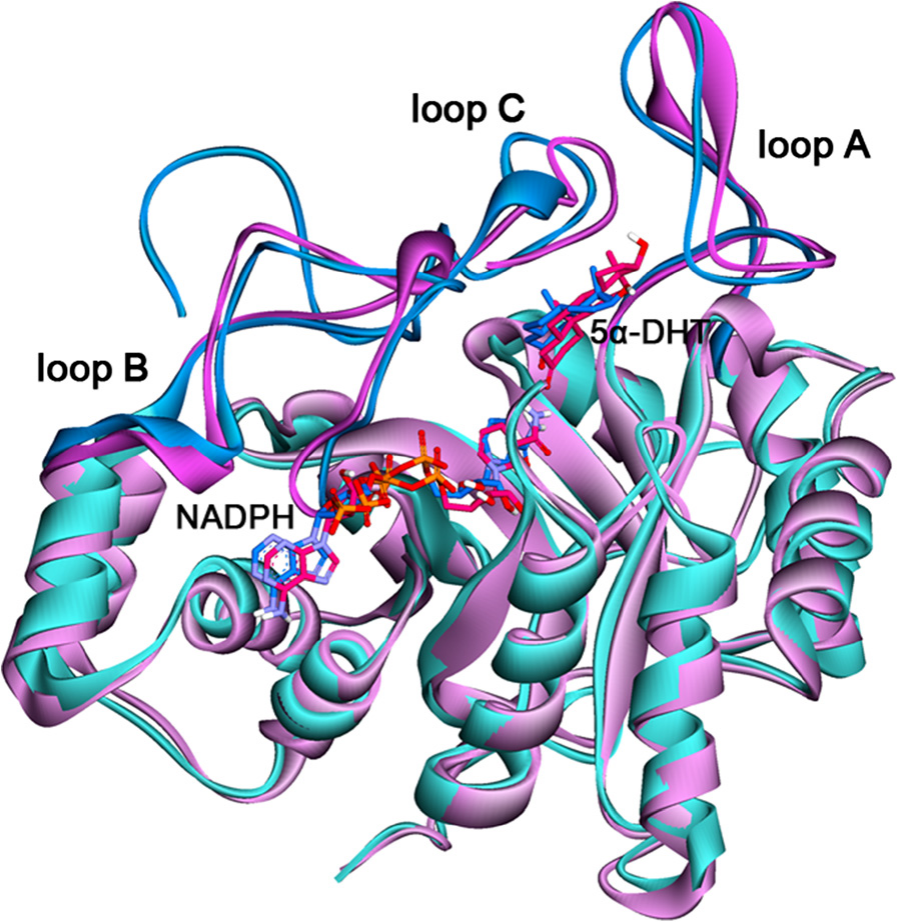
**Superposition of C-type and T-type.** The C-type was superimposed into T-type using C_α_ atoms of the proteins. The C-type (blue) and T-type (pink) are shown as cartoon models and loop regions are drawn as more dark colors. NADPH and 5α-DHT are indicated as stick models. Only polar hydrogens are shown.

**Figure 5:**
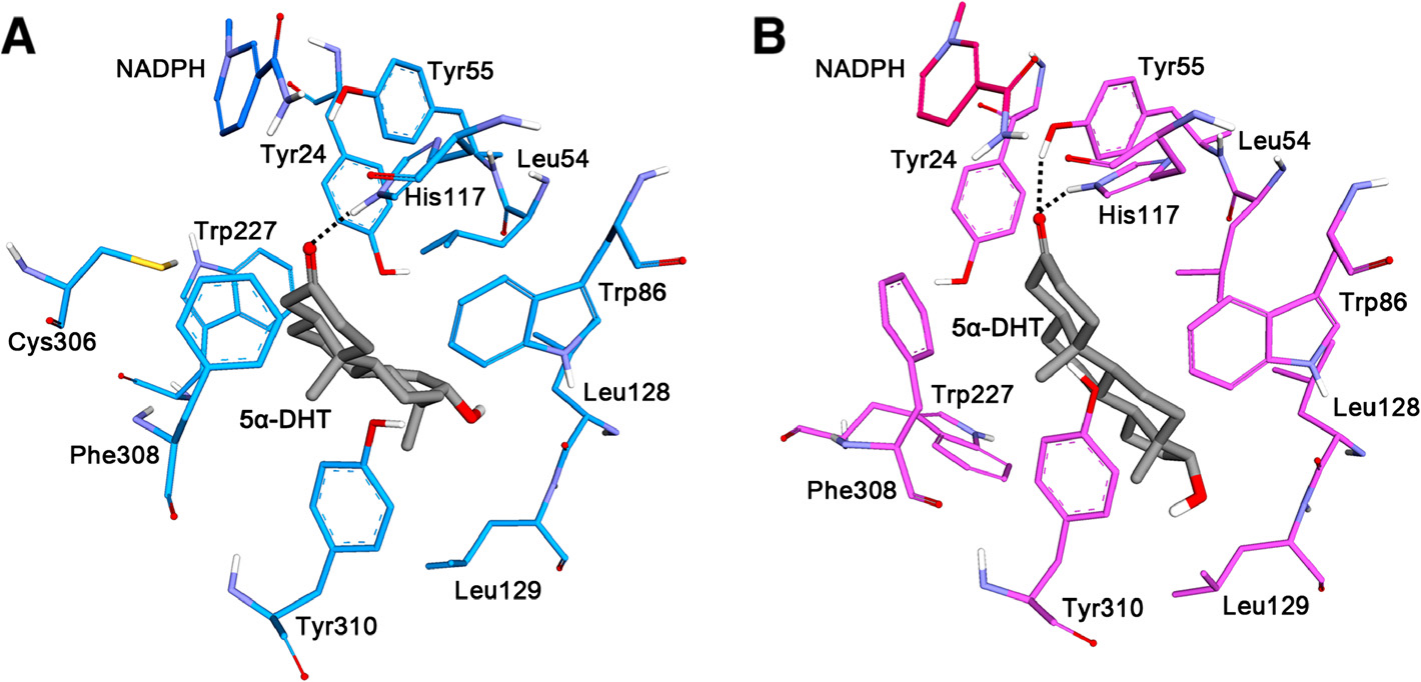
**Binding mode of 5α-DHT in the active site of AKR1C1. A** 5α-DHT binding in C-type, **B** in T-type. The C-type (blue) and T-type (pink) are depicted as cartoon models while NADPH, 5α-DHT, and the residues involving molecular interactions with 5α-DHT are shown as stick model and labeled. The hydrogen bonds are shown as black dash lines.

A molecular docking study was performed to discover proper binding conformations for 5α-DHT at the active site of the C-type and T-type. The docking conformations were clustered and ranked according to their GOLD fitness scores. A conformation having high fitness score in the most populated cluster was selected as putative binding pose of each system. The docking results revealed that 5α-DHT bound to the both types of AKR1C1 in a similar manner forming hydrophobic interactions with Tyr24, Leu54, Trp86, Phe118, Leu128, Trp227, Phe308, and Tyr310 which have been reported as key residues for steroid binding [34,35]. We found that 5α-DHT formed two hydrogen bonds with Tyr55 and His177 in the C-type, whereas, in the T-type, there was additional hydrogen bond with Leu129 as well as two hydrogen bonds. The final docking poses in C-type and T-type were used as initial structures in MD simulation study to understand the effect of C-terminal region on the enzymatic activity in atomic level. We evaluated the overall stability of MD simulations by calculating C_α_ root-mean-square deviation (RMSD), potential energy, and the number of intra-hydrogen bonds (Figure 3). The RMSD values for each system were converged to around 0.25 nm in C-type and 0.1 nm in T-type (Figure 3A). During the whole simulation time, the RMSD value of C-type was relatively higher than that of T-type with the average value of 0.23 nm and 0.11 nm, respectively. The RMSD plot for only 5α-DHT also revealed that the substrates in both C- and T-types achieved stabilization and their average values were 0.03 nm (inserted in Figure 3A). Moreover, potential energy and the number of intra-hydrogen bonds for the systems remained constant for the simulation time (Figure 3B and C). These results indicate that the MD simulations for both systems were successfully completed and there were no abnormal behaviors in the structures throughout the simulation time. A structural comparison between the C-type and T-type was performed using their representative structures which were the closest snapshot to the average of all snapshots obtained from the last 5 ns. Although there were no significant conformational changes in both systems, they showed a difference in 5α-DHT binding in terms of hydrogen bond interactions (Figure 4). The C-type showed only one hydrogen bond interaction between oxygen atom of 5α-DHT and hydrogen atom of His117 with the distance of 0.21 nm (Figure 5A). On the other hand, oxygen atoms of 5α-DHT formed hydrogen bonds with hydrogen atoms of Tyr55 and His177 in T-type and the distances of bonds were within 0.21 nm (Figure 5B). The residues Tyr24, Leu54, Trp86, Leu128, Leu129, Trp227, Phe308, and Tyr310 in both structures were participated in hydrophobic interactions which were similar to that observed in the initial docked structures. These further stabilized 5α-DHT binding in both active sites.

### Difference in 5α-DHT binding between C- type and T-type

**Figure 6:**
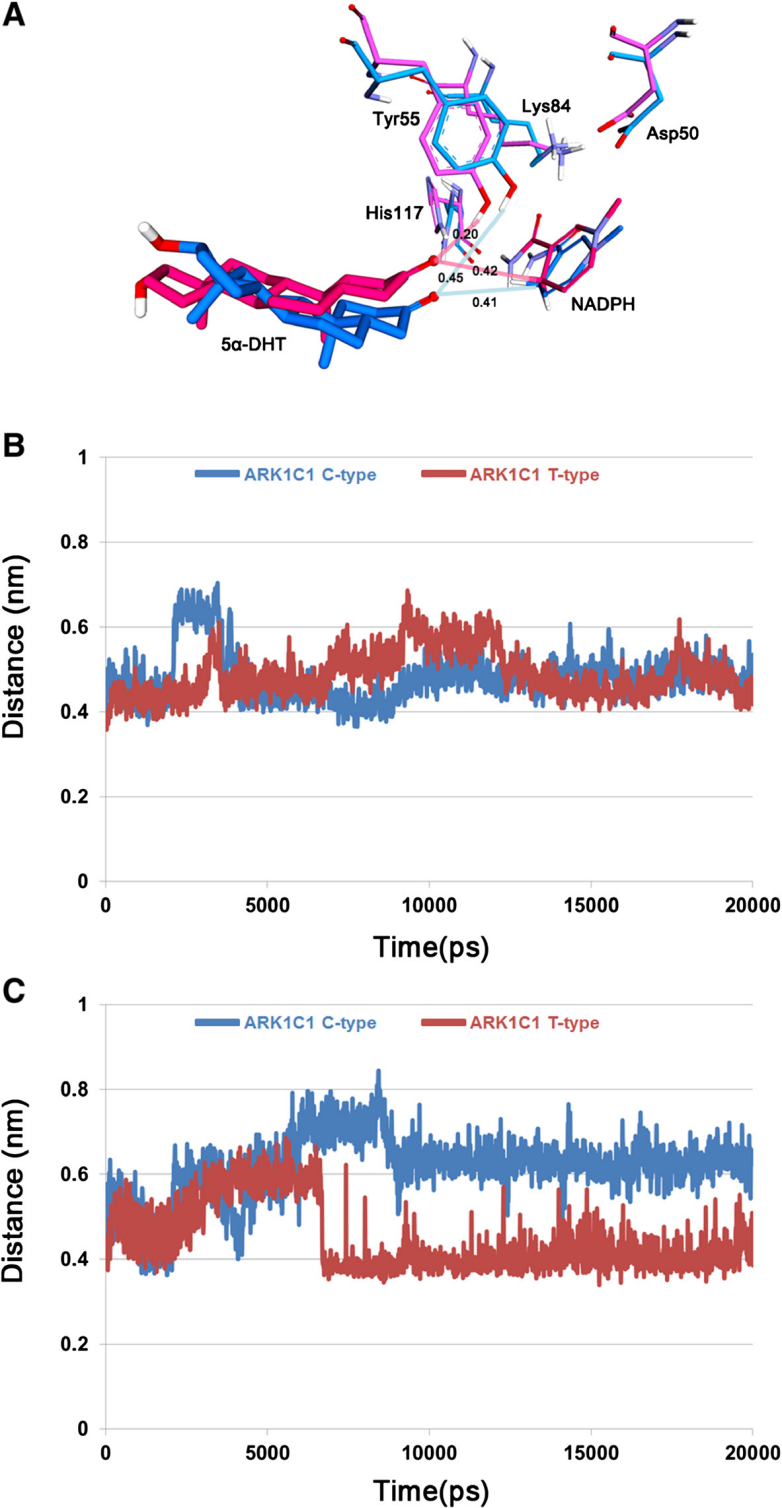
**The measurement of key distance for reduction reaction. A** The crucial distance to initiate catalytic reaction and relative position of the catalytic tetrad and cofactor in the active site of C-type and T-type. The catalytic tetrad and 5α-DHT in C-type (blue) and T-type (pink) are displayed as stick models. The key distances are given in nm. **B** The distance between C3 of 5α-DHT and C4N of NADPH. **C** The distance between C3 of 5α-DHT and OH of Tyr55. The both distances in C- and T-types were measured during 20 ns simulation time. Blue and red lines indicate C-type and T-type, respectively.

**Figure 7:**
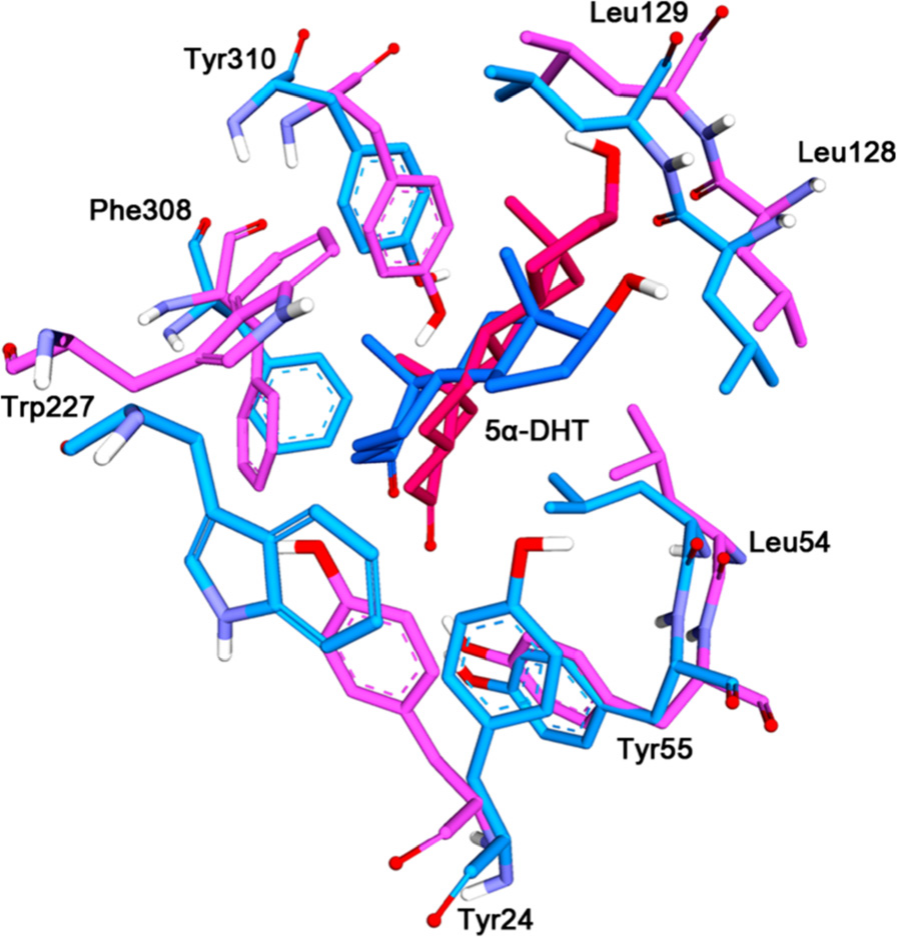
**Comparison of the 5α-DHT binding in the active site of C-type and T-type.** Interacting residues and 5α-DHT in C-type (blue) and T-type (pink) are drawn as stick models.

**Figure 8:**
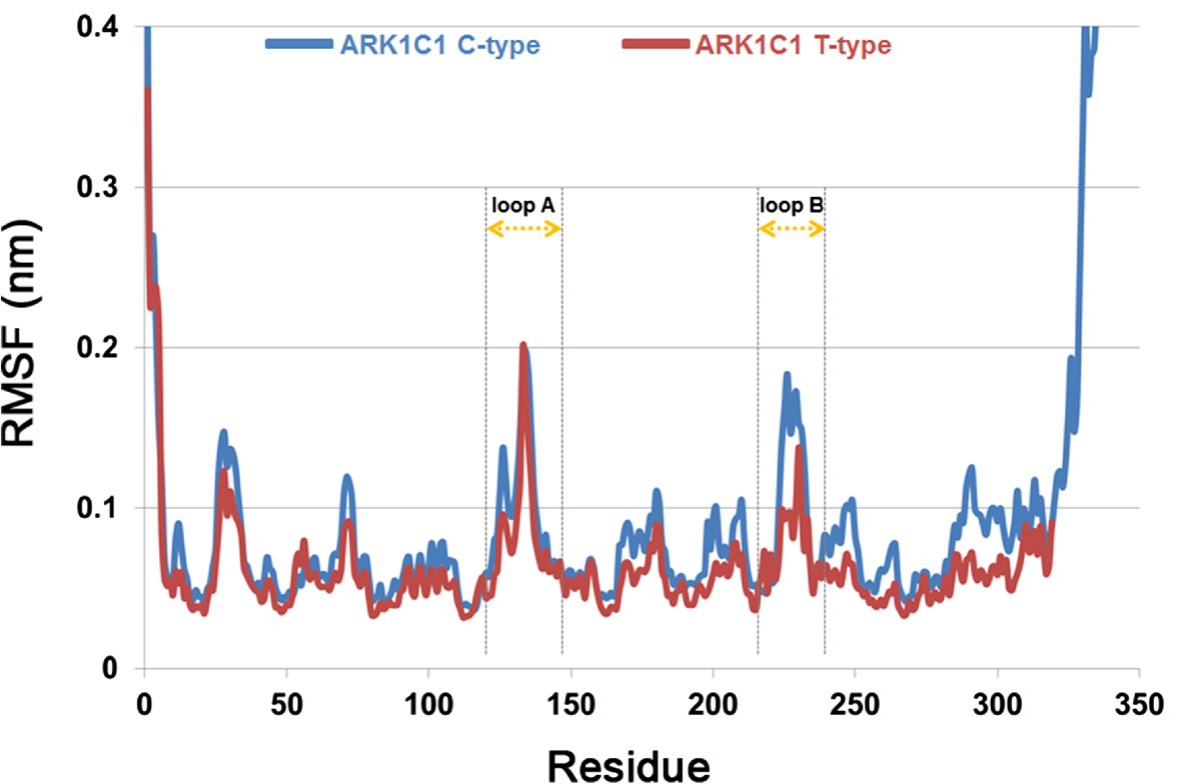
**RMSF plot showing the atomic fluctuations by residues of C-type and T-type.** RMSF values for C_α_ atoms of the proteins are drawn as blue and red lines, respectively.

**Figure 9:**
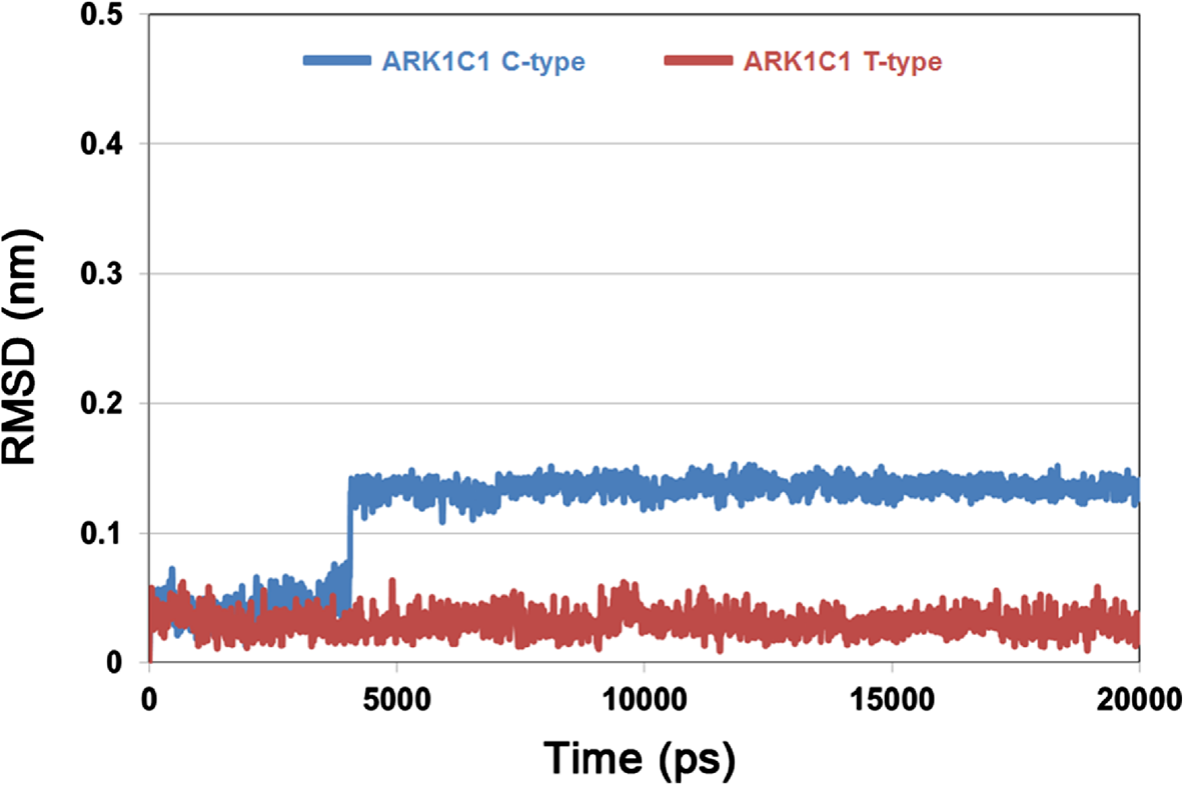
**RMSD plot for Trp227 of C-type and T-type.** RMSD values for all atoms of Trp227 are displayed as blue and red lines, respectively.

A major difference in 5α-DHT binding for C-type and T-type of AKR1C1 was the relative distance from Tyr55 which is important to initiate the catalytic reaction of the enzyme. The C3 ketone of 5α-DHT in the T-type was positioned much closer to the catalytic tetrad and the 4-pro-R hydride of the NADPH than in the C-type (Figure 6A). The distance between C3 position of 5α-DHT and C4 position in nicotinamide ring of NADPH were 0.41 nm in C-type and 0.42 nm in T-type. In contrast, the distance between the C3 of the 5α-DHT and hydroxyl group of Tyr55 in T-type was 0.20 nm which is much shorter than the value of 0.45 nm in C-type. The monitoring these distances during 20 ns simulation time revealed that the both distances were relatively short in T-type compared to C-type (Figure 6B and C). Superimposition of the two structures showed that 5α-DHT in T-type was sandwiched between Leu54 and Trp227 and its β-face was oriented toward Trp227, whereas in the case of C-type, the flipping of the side chain of Trp227 hindered the interaction with β-face of 5α-DHT (Figure 7). The side chain of Tyr24 also showed different conformation in the both types and that was probably due to displacement of Trp227. From the structural comparison, it appears that binding conformation of 5α-DHT in T-type was more favorable for catalytic reaction than that of C-type. In root mean square fluctuation (RMSF) plot, it was observed that the residues 226–229 in T-type exhibited higher flexibility than in C-type, while flexibilities of other residues were quite similar in the both structures except for highly flexible regions such as N- or C-terminal part of the protein (Figure 8). These differences might be explained by flipping of Trp227 in C-type. From RMSD plot calculated using all atoms of Trp227, the RMSD value in C-type showed relatively high with the average of 0.12 nm and it started to increase from 5 ns (Figure 9). In the simulation for C-type, the flipping of Trp227 side chain was observed and 5α-DHT was gradually alienated from Tyr55 during that time. Compared to C-type, RMSD value of Trp227 in T-type was very stable, less than 0.05 nm, throughout 20 ns simulation time and the average value was 0.03 nm. This might be related to the observation that flipping of Trp227 hardly ever happened in T-type. These analyses demonstrated that the instability of Trp227 caused by flipping of the side chain might be correlated with the distance from 5α-DHT to the hydroxyl group of Tyr55. Additionally, the interaction energy between 5α-DHT and the protein was −36.42 kcal/mol in C-type and −44.98 kcal/mol in T-type. It also indicated that 5α-DHT in T-type had energetically favorable conformation.

## Conclusions

The study of enzyme kinetics revealed that the C-terminal region in AKR1C1 contributed significantly the enzymatic properties for 5α-DHT reduction. To gain structural insights into the difference between C-type and T-type of AKR1C1 for 5α-DHT reduction, MD simulations for both structures were carried out. Prior to the simulation, we generated homology model structure for AKR1C1 due to lack of experimentally determined structures. Then C-type and T-type in complex with 5α-DHT obtained from molecular docking study were used as initial conformations for MD simulation. Although there were no significant conformational changes in both systems during 20 ns simulation time, binding conformations of 5α-DHT were different in the active site of C-type and T-type. The structural comparisons showed that T-type formed strong hydrogen bonds with Tyr55 and His117, while only His117 was found in C-type. To initiate catalytic reaction, the C3 ketone group of 5α-DHT should be close to Tyr55 and the nicotinamide ring of NADPH which are involved in hydride transfer. The distances between these groups were monitored during 20 ns simulation time. As a result, 5α-DHT was close to the cofactor in the both structures, whereas the distance between 5α-DHT and Tyr55 in T-type was relatively much shorter than C-type. On the contrary, the flipping of the side chain of Trp227 in C-type might disrupt the interaction with β-face of 5α-DHT. The interaction energies between 5α-DHT and the proteins also indicated that T-type was energetically stable compared to C-type. Taken together, our simulation results demonstrated that binding conformation of 5α-DHT in T-type was more favorable for catalytic reaction than that of C-type. These structural explanations were also in agreement with kinetic experimental results. Our findings will be useful to understand molecular mechanism for the enzyme kinetics of AKR1C1 protein.
